# Giant Lysosomes as a Chemotherapy Resistance Mechanism in Hepatocellular Carcinoma Cells

**DOI:** 10.1371/journal.pone.0114787

**Published:** 2014-12-10

**Authors:** Federico Colombo, Elena Trombetta, Paola Cetrangolo, Marco Maggioni, Paola Razini, Francesca De Santis, Yvan Torrente, Daniele Prati, Erminio Torresani, Laura Porretti

**Affiliations:** 1 Clinical Chemistry and Microbiology Laboratory, Flow Cytometry and Experimental Hepatology Service, Fondazione IRCCS Ca’ Granda Ospedale Maggiore Policlinico, Milan, Italy; 2 Clinical Pathology Laboratory, Fondazione IRCCS Ca’ Granda Ospedale Maggiore Policlinico, Milan, Italy; 3 Stem Cell Laboratory, University of Milan, Department of Pathophysiology and Transplantation, Centro Dino Ferrari, Fondazione IRCCS Ca’ Granda Ospedale Maggiore Policlinico, Milan, Italy; 4 Department of Transfusion Medicine and Hematology, Ospedale A. Manzoni, Lecco, Italy; University of Navarra School of Medicine and Center for Applied Medical Research (CIMA), Spain

## Abstract

Despite continuous improvements in therapeutic protocols, cancer-related mortality is still one of the main problems facing public health. The main cause of treatment failure is multi-drug resistance (MDR: simultaneous insensitivity to different anti-cancer agents), the underlying molecular and biological mechanisms of which include the activity of ATP binding cassette (ABC) proteins and drug compartmentalisation in cell organelles. We investigated the expression of the main ABC proteins and the role of cytoplasmic vacuoles in the MDR of six hepatocellular carcinoma (HCC) cell lines, and confirmed the accumulation of the yellow anti-cancer drug sunitinib in giant (four lines) and small cytoplasmic vacuoles of lysosomal origin (two lines). ABC expression analyses showed that the main ABC protein harboured by all of the cell lines was PGP, whose expression was not limited to the cell membrane but was also found on lysosomes. MTT assays showed that the cell lines with giant lysosomes were more resistant to sorafenib treatment than those with small lysosomes (p<0.01), and that verapamil incubation can revert this resistance, especially if it is administered after drug pre-incubation. The findings of this study demonstrate the involvement of PGP-positive lysosomes in drug sequestration and MDR in HCC cell lines. The possibility of modulating this mechanism using PGP inhibitors could lead to the development of new targeted strategies to enhance HCC treatment.

## Introduction

The resistance of tumour cells to anti-cancer agents continues to be a major cause of treatment failure in cancer patients. Multi-drug resistance (MDR) describes a situation in which cancer cells become simultaneously resistant to different drugs that have no obvious similarities in terms of structure or mechanism of action [1].

Over the last 20 years, research has revealed that MDR is multifactorial and involves decreased drug accumulation and/or increased efflux, an increased detoxification capacity, improved DNA repair, alterations in drug target susceptibility, apoptotic defects, and the induction of alternative growth factor signalling and epithelial to mesenchymal transition [1]. One of the best-characterised mechanisms of MDR occurs via cytoprotective drug pumps located into the plasma membrane that actively efflux various cytotoxic compounds [2] thus decreasing intra-cellular drug concentrations. These pumps include the ATP binding cassette (ABC) transporter family of 48 proteins that have been divided into seven sub-groups (A-G) on the basis of their sequence homology [3] and lung resistance-related protein (LRP) [4]. It has been fond that the poly-specific drug transporters ABCB1 (P-glycoprotein, PGP), ABCC1 (multidrug resistance-associated protein 1, MRP1), ABCG2 (breast cancer resistance protein, BCRP) and the ribonucleoprotein LRP are over-expressed in various types of cancer [4]–[7], and a number of studies have investigated the possibility of using conventional drugs or siRNA to inhibit ABC and LRP proteins in order to overcome MDR in myelomas and solid tumours such as ovarian, renal and hepatocellular carcinomas (HCCs) [8]–[13]. However, although promising *in vitro*, conventional drug therapies have been found to have highly toxic side effects *in vivo* due to physiological pump blockade and the competitive inhibition of cytochrome P-450 enzymes leading to increased plasma drug concentrations [14]. Second- and third-generation inhibitors have developed in an attempt to overcome these drawbacks but, although they have fewer side effects, they are also less efficacious [15].

Since the finding of MDR proteins on cell membranes, researchers have begun to investigate the role of cell compartments and organelles in the chemoresistance process and, using various MDR breast, colon, renal and ovarian cancer cell lines, a number of groups have shown that the intra-cellular compartmentalisation of anti-cancer drugs can reduce their effectiveness by limiting access to intra-cellular drug targets [16]–[18].

Similarly, we have recently demonstrated the presence in the same primary human HCC of three tumour cell clones with different degrees of *in vitro* chemoresistance [19] and, taking advantage of the yellow colour of sunitinib, noticed that the most drug-resistant cell clone (Hcc-1) showed drug accumulation in intra-cellular vacuoles during *in vitro* culture.

The aim of this study was to investigate the nature of these drug-accumulating vacuoles and their possible role in the process of drug resistance, and we have observed that tyrosine kinase inhibitors (TKIs) - including sorafenib, the only oral drug approved for the treatment of advanced HCC - accumulate in cell lysosomes and documented the fact that this can influence the chemosensitivity of HCC cells.

## Materials and Methods

### Cell cultures

Five commercial human HCC cell lines (HuH7, HepG2, Hep3B, PLC/PFR/5 and SNU475), purchased from the Japanese Collection of Research Bioresources (JCRB) or the American Type Cell Collection (ATCC), and one primary HCC cell line, obtained in our laboratory (Hcc-1) [19], were cultured in IMDM+GlutaMAX supplemented with 10% FBS, 1% penicillin-streptomycin and 1% non-essential amino acids (Invitrogen, Life Technologies, Milan, Italy) on collagen type I-coated flasks or multi-well plates. The cells were maintained at 37°C in a humidified incubator containing 5% CO_2_.

### Sunitinib accumulation

In order to verify drug absorption, the cells were incubated with sunitinib (formerly SU11248, Pfizer, New York, NY, USA), which can be easily visualised in culture because of its yellow colour and green fluorescence. The cells were kept in culture with sunitinib 12 µM for two hours and the drug’s localisation was evaluated by means of microscopy.

### Vesicle localisation

The intra- or inter-cellular localisation of the vesicles with sunitinib accumulation was determined by means of immunostaining with a primary monoclonal antibody against the β1 integrin (CD29, Becton Dickinson, Franklyn Lakes, NJ, USA), which is expressed by all the studied cell lines, for one hour at 37°C. After washing, the cells were stained with a secondary anti-mouse TRITC antibody diluted 1∶50 for 30 minutes at 37°C, and then 10 µg/ml of Hoechst 33342 (Sigma-Aldrich, St.Louis, MO, USA) was added for 20 minutes for nuclear staining. The cell images were acquired and analysed using a Leica DM IRE2 fluorescence microscope (Leica Microsystem, Wetzlar, Germany).

### Oil-red-O staining

In order to exclude the possibility that the vesicles were mere lipid droplets accumulating sunitinib by diffusion, we used Oil-red-O staining. Cells cultured in 6-well plates were fixed with 10% formalin for one hour at room temperature (RT) and washed three times with 60% 2-propanol (Carlo Erba, Milan, Italy). After drying, 2 mg/mL of Oil-red-O (Sigma-Aldrich) in 2-propanol was added for 10 minutes at RT, the cells were washed four times with distilled water, and images were acquired using an Eclipse TS100 microscope (Nikon, Tokyo, Japan).

### Sunitinib accumulation after weak base cell treatment

To test the lysosomal origin of cell vesicles, we re-evaluated drug distribution in the presence of ammonium chloride (NH_4_Cl) in culture medium. In fact, weak bases, buffering the lysosomal pH, are able to inhibit the accumulation of cationic amphiphilic drugs (such as sunitinib) in lysosomes [17]. Cells were seeded in a 24-well plate and treated as follow:

Well 1) NH_4_Cl 3 mM for 30 minutes followed by sunitinib 12 µM+NH_4_Cl 3 mM for further 90 minutes.

Well 2) sunitinib 12 µM for 60 minutes followed by sunitinib 12 µM+NH_4_Cl 3 mM for further 90 minutes.

Cells were monitored by means of optical microscopy and pictures were taken before treatment, and after the first and second incubation.

### Immunofluorescence staining of cell vesicles

Tumour cells were directly fixed with 70% ethanol in 96-well plates, permeabilised and blocked with a solution of 10% goat serum and 0.1% Tween 20 (Sigma-Aldrich) for 20 minutes at RT, and then stained with polyclonal rabbit anti-vacuole membrane protein 1 (VMP1), anti-human peroxisomal membrane protein 70 (PMP70), monoclonal mouse anti-protein disulfide isomerase (PDI) (all from Millipore, Billerica, MA, USA) or anti-lysosome-associated membrane protein-1 (LAMP-1) (Becton Dickinson) for two hours at RT. After washing, the cells were incubated with FITC-conjugated goat anti-rabbit or anti-mouse antibody for one hour in the dark at RT and, after further washing, DAPI was added for nuclear staining. The images were acquired and analysed using a Leica DM IRE2 microscope.

### Western Blot

Whole cell extracts were prepared using a lysis buffer containing 20 mM Tris-HCl (pH 7.8), 140 mM NaCl, 1 mM EDTA, 0.5% NP40, 1 mM phenylmethylsulfonil fluoride, and complete protease inhibitor mixture (Roche Diagnostics, Mannheim, Germany). Cell lysates were first passed 5 times through a 30.5-gauge needle to disrupt the nuclei, then incubated at 4°C for 15 minutes and finally centrifuged at 13000 rpm for 15 minutes at 4°C. Total protein concentration was determined according to Lowry’s method [20].

Samples were resolved on 8% polyacrylamide gel, transferred to supported nitrocellulose membranes (Bio-Rad Laboratories, Hercules, CA, USA), and the filters were saturated in blocking solution (10 mM Tris (pH 7.4), 154 mM NaCl, 1% BSA, 10% horse serum, and 0.075% Tween-20). The anti-human LAMP-1 (1∶250) (Millipore, Darmstadt, Germany) antibody was incubated overnight at 4°C. We also determined the expression of the housekeeping protein β-Tubulin III (Sigma-Aldrich) in each sample. Detection was performed with horseradish peroxidase (HRP)-conjugated secondary antibodies (Dako Cytomation, Carpinteria, CA, USA), followed by enhanced chemiluminescence (ECL) development (Amersham Biosciences, Piscataway, NJ, USA). Pre-stained molecular weight markers (Bio-Rad Laboratories, Hercules, CA, USA) were run on each gel. Bands were visualized by autoradiography using Amersham Hyperfilm Images of bands were obtained using the CanoScan LiDE60 Scanner (Canon, Milan, Italy) and the Canon ScanGear Software. Densitometric analyses were performed using ImageJ software (http://rsbweb.nih.gov/ij/).

### Time-lapse experiments

Time-lapse experiments were used to follow the vesicles’ kinetics and fate. The cells were incubated with sunitinib 12 µM in complete medium (without phenol red) in a 35 mm diameter Petri dish for two hours in order to allow the cells to store the drug in their vesicles, and were then transferred to a Nikon Biostation IM. Pictures were taken every 10 minutes for seven hours.

### Flow cytometry

Tumour cells were detached from the flasks using TripLE Express (Invtrogen), resuspended and incubated in PBS supplemented with 4% fetal calf serum, 4% mouse serum and 0.1% NaN_3_ for 20 minutes at 4°C in order to saturate the unspecific bound sites, and were then stained with mouse anti-ABCG2, anti-LRP, anti-MRP1 and anti-PGP antibodies for 20 minutes at 4°C (Becton Dickinson); after washing, a secondary PE-conjugated goat-anti-mouse antibody (GAM-PE, Becton Dickinson) was added for 15′ in the dark at 4°C. In order to exclude dead cells from the analysis, 7-aminoactinomycin D (7-AAD, Becton Dickinson) was added to each tube.

The cells were immunophenotyped as previously described [19]. Briefly, they were stained with primary fluorochrome-conjugated EpCAM, CD133, CD44, CD56 and CD90 antibodies, and the data were acquired and analysed using a FACSCantoII flow cytometer and FACSDiva software (Becton Dickinson).

### Fluorescence microscopy

Cultured cells were stained in 96-well plates using the same primary monoclonal antibodies as those used for the flow cytometry analysis (anti-ABCG2, LRP, MRP1 and PGP) following the protocol for cell organelle staining. The wells were mounted, and the images taken using a Leica DM IRE2 microscope.

### Chemoresistance assays

Cell line chemoresistance was evaluated using sorafenib (LC Laboratories, Woburn, MA, USA) or sunitinib in combination with the PGP inhibitor verapamil [21] at a concentration of 20 µM, the highest non-toxic concentration on the basis of previous titration assays (data not shown).

In order to quantify sorafenib resistance, the cells underwent an MTT (3-(4,5-dimethylthiazol-2-yl)-2,5-diphenyltetrazolium bromide, Sigma-Aldrich) assay in triplicate. The cells were seeded in 96-well plates at a concentration of 8000 cells/well in complete medium, and allowed to recover for one night at 37°C. Sorafenib was administered as follows:

1. Sorafenib 0–50 µM alone for 24 hours.
2. One hour of pre-incubation with verapamil 20 µM followed by 24 hours of co-incubation with verapamil 20 µM and sorafenib 0–50 µM.
3. One hour of pre-incubation with sorafenib 0–50 µM followed by 24 hours of co-incubation with verapamil 20 µM and sorafenib 0–50 µM.

After drug treatment, the cells were incubated with 5 mg/ml MTT solution for four hours at 37°C and, after medium removal, the MTT salts were dissolved with DMSO for five minutes; viability was evaluated on the basis of 550 nm absorption using a Xenius plate reader (Safas, Munich, Germany). IC_50_ values were calculated by means of non-linear regression analysis using GraphPad Prism version 5.0 software (GraphPad Software, San Diego, CA, USA).

Moreover, we implemented another MTT assay on HepG2 and SNU475, as representative cell lines with giant and small lysosomes, using NH_4_Cl 3 mM instead of verapamil with the same scheme reported above.

Similarly, all cell lines cultured in 24-well plates were treated with sunitinib 12 µM (corresponding to the lowest detected IC_50_) using the same treatment protocols as those used for sorafenib in order to monitor cell death visually. The cells were monitored by means of optical microscopy and pictures were taken before treatment, after pre-incubation, and after two and 12 hours of treatment.

### Statistical analyses

The data were statistically analysed using Friedman’s test or Student’s t test as required, and GraphPad Prism version 5.0 software. P values of <0.05 were considered statistically significant.

## Results

### Sunitinib accumulation

**Figure 1:**
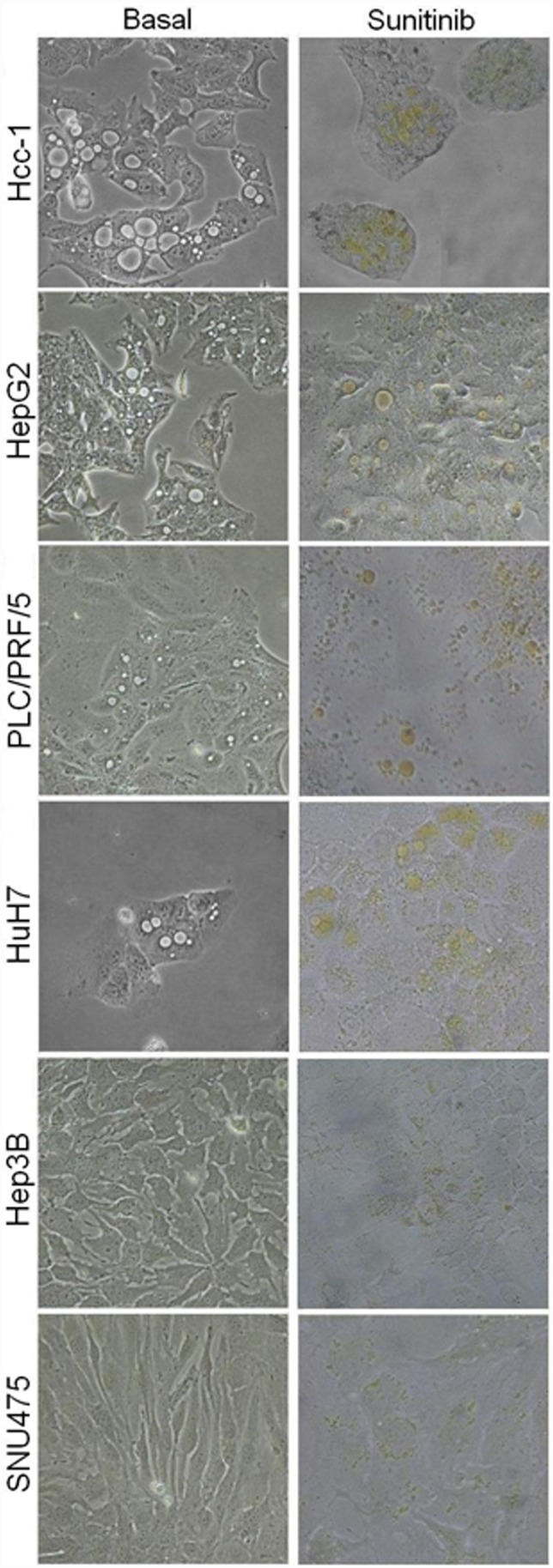
Sunitinib accumulation in cytoplasmic vesicles of HCC cell lines. Representative pictures of cell lines cultured without (left) or with (right) sunitinib 12 µM. Cytoplasmic vesicles are visible in the Hep3B and SNU475 cells only after sunitinib incubation. Original magnification 20x.

Under basal conditions, the HCC cell lines showed different patterns of vesicle expression: the HepG2 and Hcc-1 lines had 1–2 large cytoplasmic vacuoles per cell; the HuH7 and PLC/PRF/5 lines had 1–2 medium-sized vesicles; and the Hep3B and SNU475 had no visible vesicles. After sunitinib incubation, large yellow vesicles were visible in the HepG2, Hcc-1, HuH7 and PLC/PRF/5 cells, whereas the Hep3B and SNU475 cells showed only very small yellow cell vesicles (Fig. 1).

### Vesicle localisation

**Figure 2:**
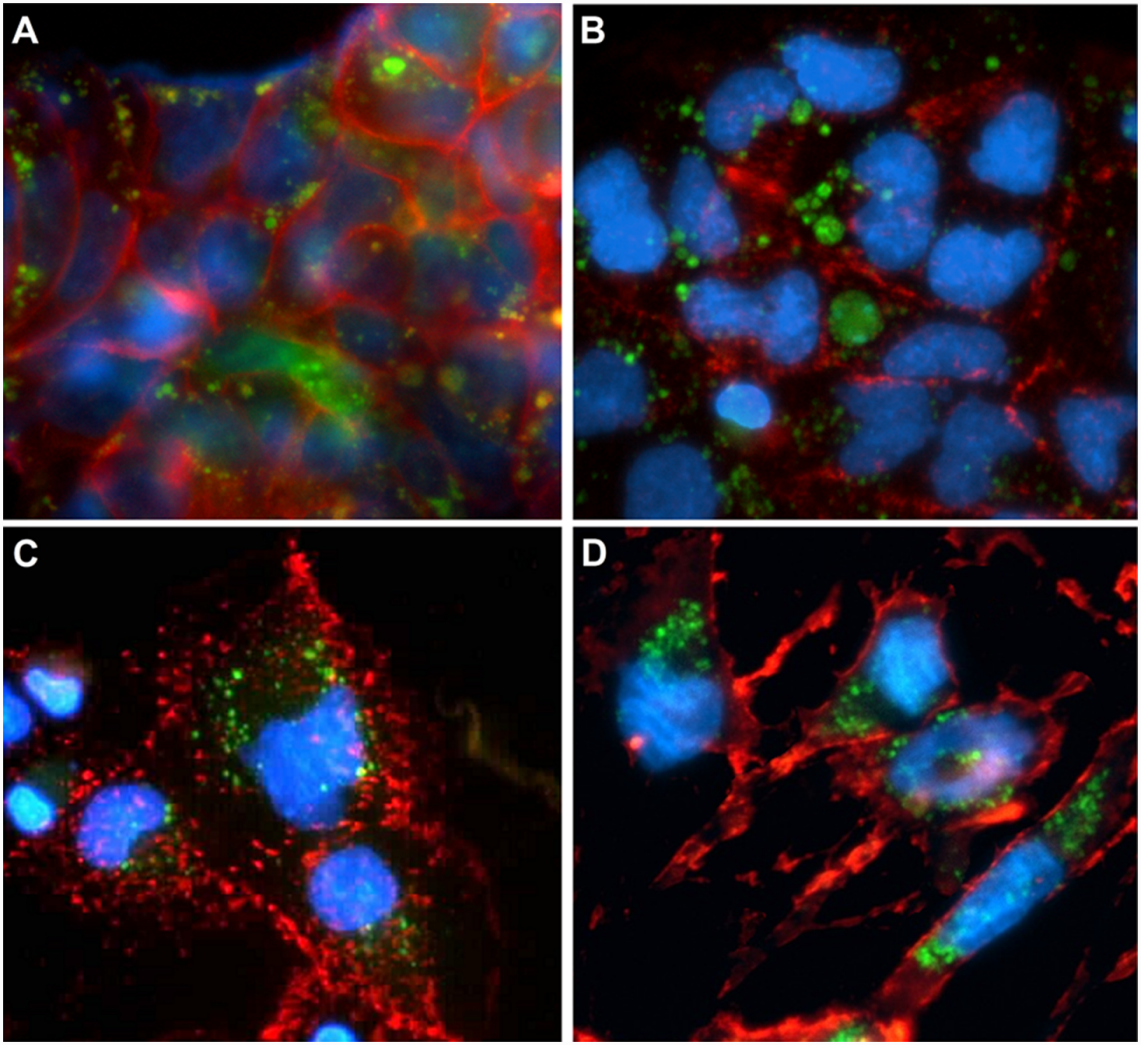
Cytoplasmic vesicle localisation. Fluorescence microscopy images confirming intra-cellular vesicle localisation. (A) Hcc-1, (B) HepG2, (C) Hep3B, and (D) SNU475 cells. Sunitinib autofluorescence (green), CD29 staining (red) and Hoechst 33342 nuclear staining (blue). Original magnification 40x.

**Figure 3:**
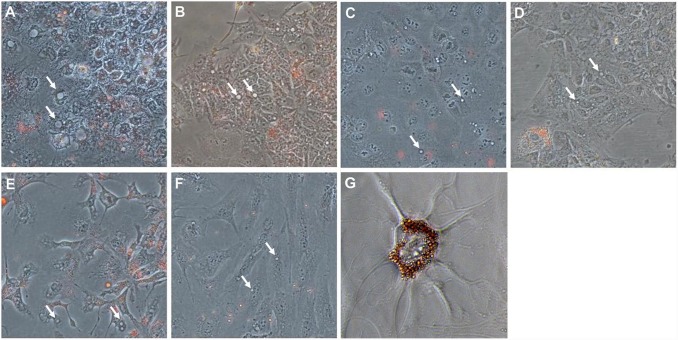
Oil-red-O staining. The cytoplasmic vesicles of all of the cell lines were negative for Oil-red-O staining (white arrows), ruling out that are lipid droplets. (A) Hcc-1, (B) HepG2, (C) PLC/PRF/5, (D) HuH7, (E) Hep3B, (F) SNU475, and (G) bone marrow adipocyte (positive control). Original magnification 20x.

Fig. 2 clearly shows the intra-cellular localisation of green vesicles (sunitinib autofluorescence) after staining the cytoplasmic membranes of HCC cells with β1 integrin. No Oil-red-O staining of the cytoplasmic vesicles in any of the cell lines was observed (Fig. 3), thus ruling out the possibility that they were mere lipid droplets. Furthermore, cell pre-incubation with NH_4_Cl did not allow sunitinib accumulation in cell vesicles, while sunitinib-loaded vesicles released the drug after NH_4_Cl addition in culture medium, thus suggesting a lysosomal origin of cell vesicles (S1 Figure). Moreover, we also observed an increased number of lysosomes in Hcc-1, HepG2, PLC/PRF/5 and HuH7 after NH_4_Cl incubation.

### Immunofluorescence staining, Western blot and time-lapse experiments

**Figure 4:**
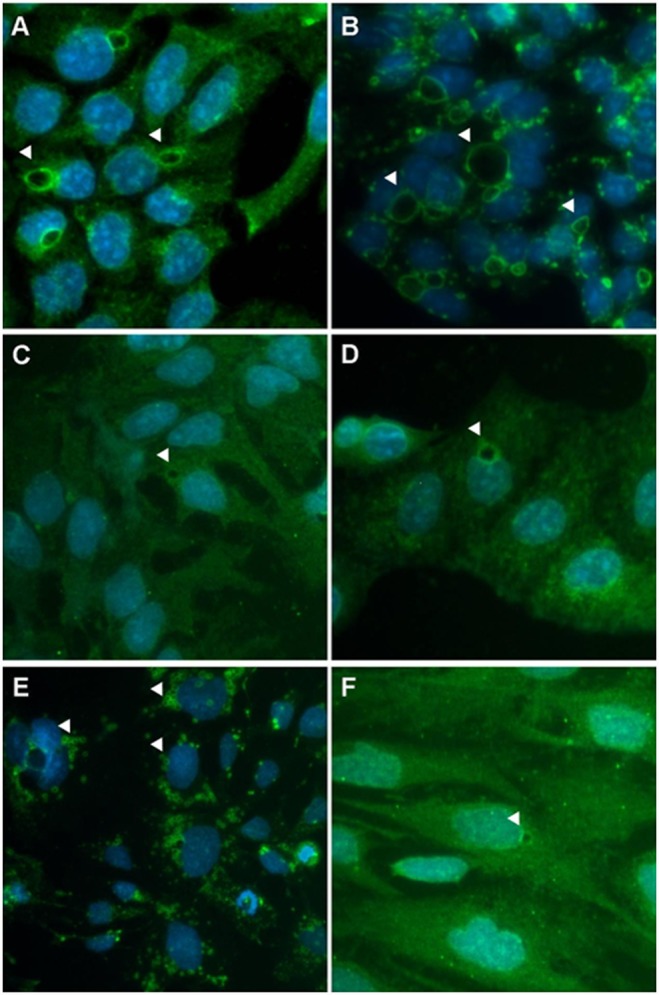
Immunofluorescence staining of cell organelles. Vesicle membranes (arrowheads) stained positive for anti-LAMP1 antibody (green). All of the other antibodies against organelle proteins were negative (data not shown). (A) Hcc-1, (B) HepG2, (C) PLC/PRF/5, (D) HuH7, (E) Hep3B, and (F) SNU475. Nuclei were stained with DAPI (blue). Original magnification 20x.

**Figure 5:**
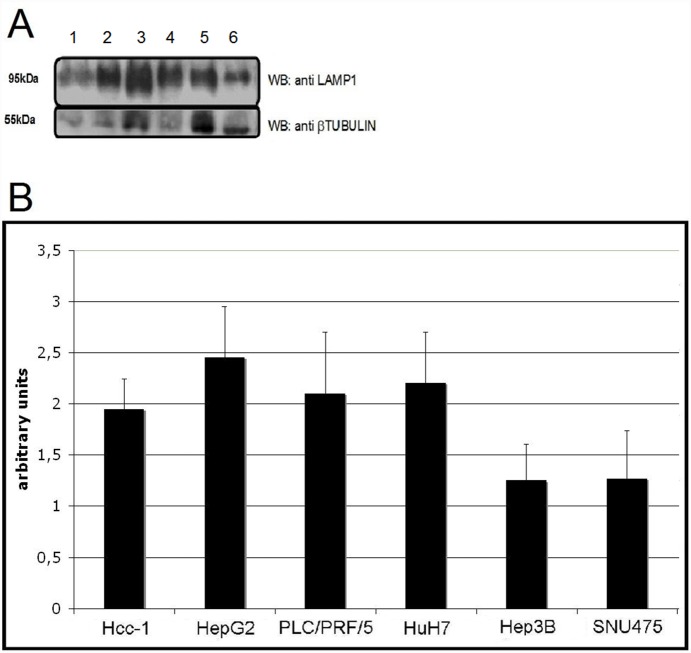
Western blot. A) Whole cell extracts were analyzed for LAMP-1 protein expression levels by Western blot using β tubulin as loading control. 1) Hcc-1, 2) HepG2, 3) PLC/PRF/5, 4) HuH7, 5) Hep3B and 6) SNU475. B) Relative quantification of LAMP-1 expression. The four HCC cell lines carrying giant lysosomes (Hcc-1, HepG2, PLC/PRF/5 and HuH7) showed higher LAMP-1 expressions as compared to Hep3B and SNU475 (showing small lysosomes).

The intra-cellular vesicles stained positively for LAMP-1 (Fig. 4) but negatively for VMP1, PMP70 and PDI (data not shown), thus indicating their lysosomal nature. Western blot analysis confirmed a higher expression of LAMP-1 in those cell lines carrying giant lysosomes as compared to those characterized by small lysosomes (p<0.05) (Fig. 5).

**Figure 6:**
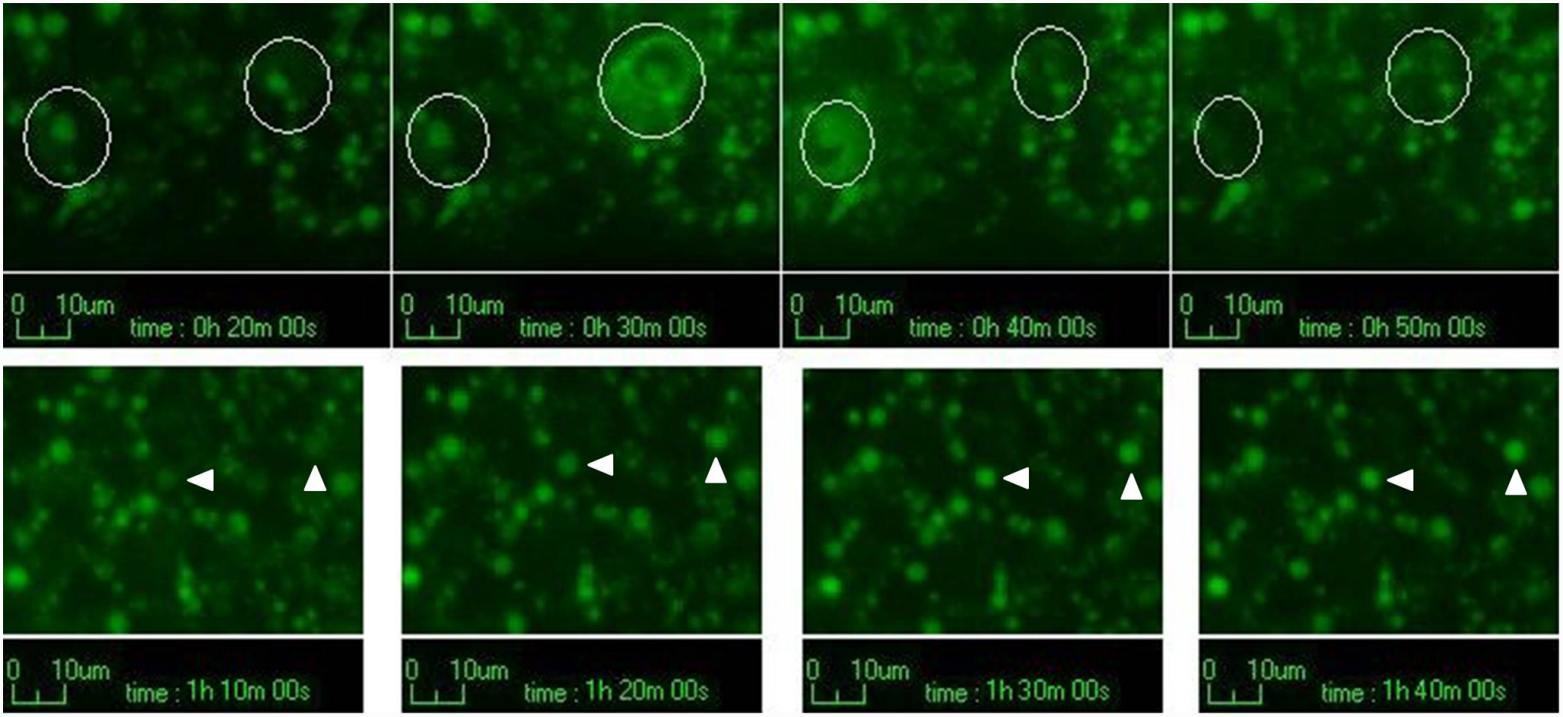
Time-lapse experiments. Over a period of seven hours it is possible to see that some lysosomes released their content outside the cells (top row, white circles), whereas others started to accumulate new sunitinib in their lumens (bottom row, white arrowheads).

The time-lapse experiments showed that some of the vesicles retained their content for seven hours, whereas others fused with the cell membrane and released their contents outside the cells. This behaviour is consistent with that of secretory lysosomes [22]. It was also possible to observe that some of these lysosomes stored new sunitinib, thus enhancing their fluorescence (Fig. 6 and S1 Video).

### MDRP expression and cell immunophenotyping

**Figure 7:**
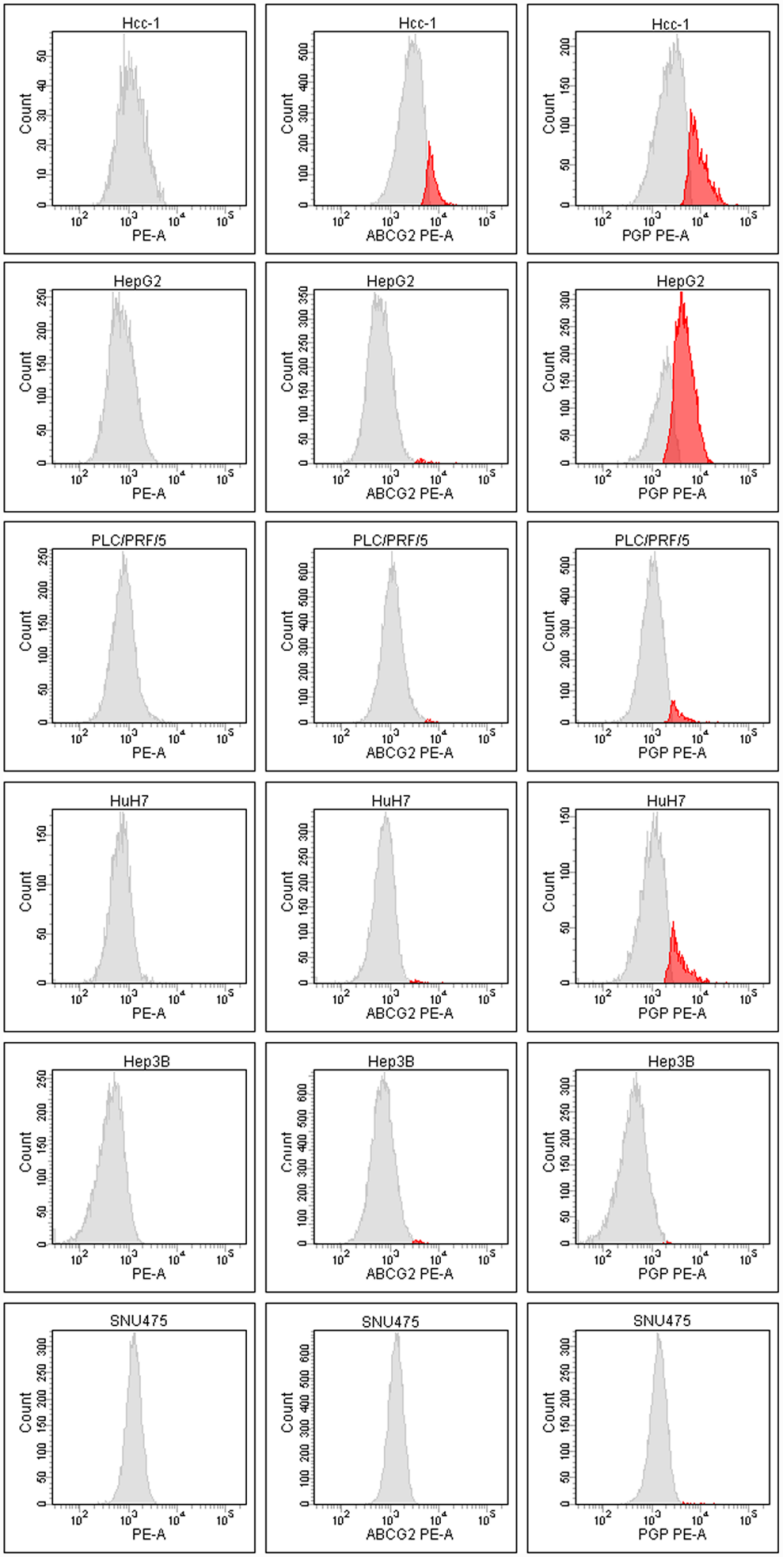
Flow cytometry evaluation of MDRPs. LRP and MRP1 were not detected by means of flow cytometry in any cell line. ABCG2 was expressed on only 9% of the Hcc-1 cells, and was almost absent on the surface of the other cell lines. ABCB1 (PGP) was highly expressed on the plasma membrane of HepG2 (58%), Hcc-1 (18%) and HuH7 cells (13%), but expressed on only about 3% of PLC/PRF/5 cells. Left column negative controls; middle column ABCG2 expression; right column PGP expression.

**Figure 8:**
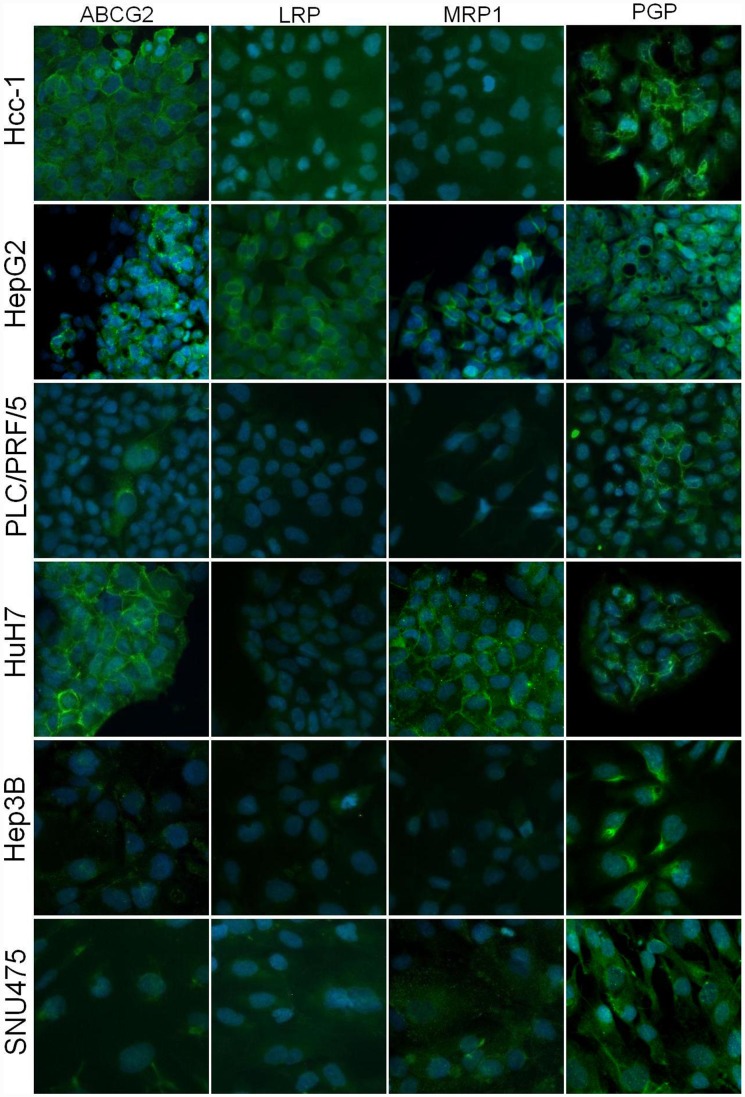
Immunofluorescence staining of MDRPs. Representative pictures of MDRP staining in HCC cell lines. The expression and localisation of MDRPs was different in the various HCC cell lines: ABCG2, LRP and MRP1 were localised on the membrane of the few groups of cells on which they were expressed, whereas PGP was mainly found in the cell cytoplasm, particularly on the membrane of intra-cellular vesicles. Nuclei stained with DAPI (blue). Original magnification 20x.

By means of flow cytometry, we observed that ABCG2 was expressed on the membrane of only 9% of Hcc-1 cells, and was almost absent from the surface of the cells in the other lines. ABCB1 was highly expressed only on the surface of HepG2 (58%), Hcc-1 (18%) and HuH7 cells (13%), but expressed on only about 3% of PLC/PRF/5 cells. The other MDRPs were expressed at levels of <2% (Fig. 7). Immunofluorescence staining revealed that, when expressed, ABCG2, LRP and MRP1 were localised at cell membrane level, whereas PGP (ABCB1) also stained lysosomes (Fig. 8).

Immunophenotyping revealed higher levels of the epithelial stem cell markers EpCAM and CD133 on Hcc-1, HepG2, Huh7 and PLC/PRF/5 cells, whereas SNU475 had high levels of the mesenchymal markers CD44, CD56 and CD90. Hep3B cells had an intermediate phenotype (Table 1).

**Table 1:** Immunophenotyping.

Marker	Hcc-1	HepG2	PLC/PRF/5	HuH7	Hep3B	SNU475
EpCAM	90	80	10	90	50	1
CD133	1	14	70	60	2	9
CD44	33	3	2	4	10	98
CD56	5	1	1	4	14	99
CD90	1	1	3	5	2	99

The table shows the mean percentage of cells positive for each marker.

### Chemoresistance assays

Different treatments with sunitinib and sorafenib in combination with verapamil were tested in order to define the best *in vitro* treatment model.

**Figure 9:**
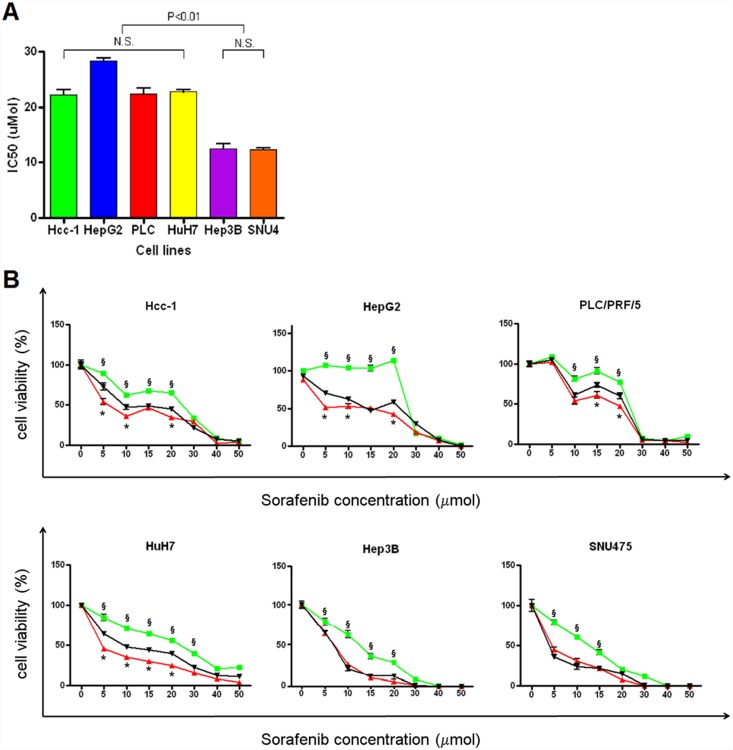
Chemoresistance assays. A) When treated with sorafenib, the cell lines with giant PGP-positive lysosomes (Hcc-1, HepG2, PLC/PRF/5 and HuH7) showed higher IC_50_ values than those with normal lysosomes (Hep3B and SNU475) (p<0.01). B) The HCC cell lines were incubated with different concentrations of sorafenib in order to verify their chemosensitivity (green lines). The cells with larger cytoplasmic vesicles were characterised by a curve with a sort of plateau of viability at sorafenib concentrations of between 5 and 20 µmol. One hour of verapamil pre-treatment used to inhibit ABC proteins before co-incubation with sorafenib and sunitinib increased the chemosensitivity of all of the cell lines (black curves). *p<0.01 green vs. black curves. One hour of sorafenib pre-treatment (red lines) before co-incubation with sorafenib and sunitinib enhanced treatment efficacy in comparison with verapamil pre-treatment in the cell lines carrying giant lysosomes. ^§^p<0.05 red vs. black curves.

Interestingly, the cell lines with giant lysosomes (Hcc-1, HepG2, PLC/PRF/5 and HuH7) had higher IC_50_ values than the lines with small lysosomes (Hep3B and SNU475) (Fig. 9A). As expected, pre-incubation with verapamil increased the efficacy of sorafenib in comparison with sorafenib alone in all of the cell lines (P<0.05) (Fig. 9B). However, unexpectedly, pre-treatment with sorafenib was more efficacious than pre-treatment with verapamil in the cell lines with giant lysosomes (P<0.05), whereas no difference was observed in the Hep3B and SNU475 cell lines (Fig. 9B).

Regarding experiments in which we substituted verapamil with NH_4_Cl, we observed a lower IC50 in cell cultures pre-incubated with both NH_4_Cl or sunitinib, whereas, differently from “verapamil experiments”, no differences were detected between the two pre-treatment schemes (S2 Figure).

**Figure 10:**
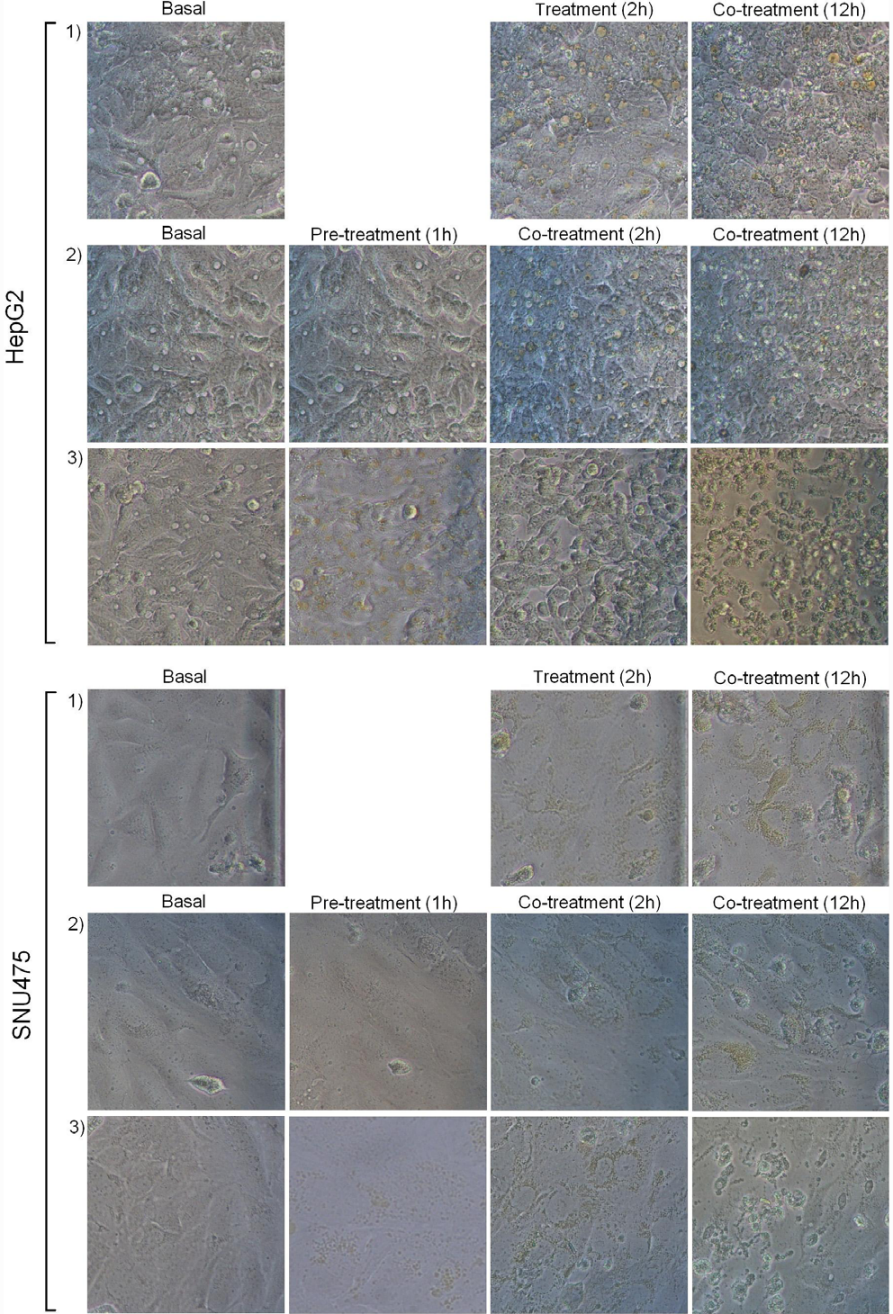
Sunitinib treatments. Representative pictures of the effect of sunitinib treatments on the HepG2 (with giant lysosomes) and SNU475 cell lines (with normal lysosomes). The rows show the treatment conditions: 1) Sunitinib 12 µM alone for 12 hours; 2) One hour of pre-incubation with verapamil 20 µM followed by the co-incubation of verapamil 20 µM and sunitinib 12 µM for 12 hours; 3) One hour of pre-incubation with sunitinib 12 µM followed by the co-incubation of verapamil 20 µM and sunitinib 12 µM for 12 hours. It can be seen that, in both cell lines, cell death after 12 hours’ treatment was greater in the cells pre-treated with sunitinib.

Finally, 12-hour cell mortality, monitored by optical microscopy, was greater in the cells pre-incubated with sunitinib than in those pre-incubated with verapamil (Fig. 10).

## Discussion

Drug resistance, which involves several different pathways, is the main mechanism responsible for treatment failure and death among cancer patients, but its underlying molecular and biological mechanisms are still unclear. However, during a recent study [19], we observed that, although generated from the same primary tumour, different HCC cell lines were differently sensitive to the tyrosine kinase inhibitor sunitinib and that this sensitivity directly correlated with the membrane expression of the drug’s target. We also observed that the highly resistant primary cell line Hcc-1 showed giant yellow vesicles in the cytoplasm after incubation with sunitinib, which is auto-fluorescent, thus indicating drug accumulation. A similar finding has been more recently reported by Date *et al*. [23], who demonstrated the accumulation of tamoxifen in the intra-cytoplasmic vacuoles of HepG2 cells. We therefore investigated the nature of these organelles and their role in inducing drug-resistance in six HCC cell lines routinely cultured in our laboratory.

During *in vitro* HCC cell culture in the presence of sunitinib, the cell lines with more epithelial characteristics (i.e. Hcc-1, HepG2, PLC/PRF/5 and HuH7) developed giant intra-cellular vacuoles, whereas those with mesenchymal features (i.e. Hep3B and SNU475) presented small vacuoles. Furthermore, these vacuoles had a lysosomal origin and intra-cellular localisation, unlike that recently described in breast cancer cells in which chemoresistance to mitoxantrone was associated with an extra-cellular localisation of vesicles [16]. However, our findings are in line with those of Gotink e*t al.* and Yamagishi *et al.*, who have described the involvement of lysosomes in drug sequestration due to the MDRP activity in renal and colon cancer cell lines [17], [18]. Moreover, as already observed by Goldman *et al*., we found an increment of the number and dimension of lysosomes after NH_4_CL incubation due to the extensive ion-trapping-based accumulation of lysosomotropic weak bases in these organelles [24].

With regard to MDRPs, our flow cytometry and fluorescence microscopy analyses revealed that ABCG2, ABCC1 and MVP were rarely expressed and only on the plasma membrane of the HCC cell lines, thus suggesting their involvement in the classic mechanism of drug resistance that involves drug expulsion from the cytoplasm to extra-cellular space. The P-glycoprotein (PGP-ABCB1) was also present on both giant and small lysosomes, thus supporting the involvement of PGP in lysosome-based drug sequestration in HCC cells.

Interestingly, we found a direct correlation between the size of the PGP-positive lysosomes and resistance to sorafenib or sunitinib treatment: the Hcc-1, HepG2, PLC/PRF/5 and HuH7 cell lines, harbouring giant lysosomes and expressing higher level of LAMP-1, showed statistically significant higher IC_50_ values than the SNU475 and Hep3B cell lines, harbouring small lysosomes and a lower level of LAMP-1. As expected, treatment with verapamil, a PGP inhibitor, enhanced the chemosensitivity of all of the cell lines to tyrosine kinase inhibitors.

**Figure 11:**
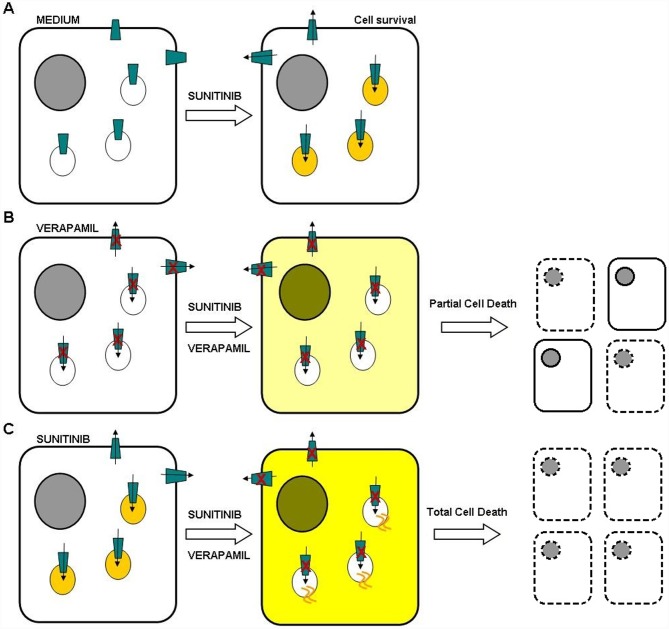
Hypothesised mechanism of the enhanced efficacy of drug pre-treatment before verapamil administration and PGP blockade. A) HCC cells expressing active PGP can expel a drug (e.g. sunitinib) from the cytoplasm or store it in lysosomes. B) Blocking PGP with verapamil before the co-administration sunitinib and verapamil allows the drugs to enter the cell and diffuse into cytoplasm/nucleus. C) If sunitinib is used for pre-treatment, it is stored in giant lysosomes and, after the co-administration of sunitinib and verapamil and subsequent PGP blockade, the drugs can enter the cytoplasm/nucleus from both extra-cellular space and the lysosomes.

Unexpectedly, pre-incubation with sorafenib before co-incubation with verapamil increased drug efficacy in the HCC cell lines with giant lysosomes. We hypothesise that, during the pre-incubation phase, anti-cancer drugs are trapped in PGP-positive lysosomes and that blocking PGP activity by means of subsequent drug/verapamil co-incubation would allow drug diffusion from the culture medium and lysosome into the cytoplasm, thus increasing intra-cellular drug concentrations (Fig. 11). On the contrary, the pre-treatments with both sunitinib or NH_4_CL showed the same increment in drug efficacy as compare to drug alone. This could be due to the absence of verapamil action on cell membrane PGP activity which leads to drug accumulation in the cytoplasm with a strong impact on cell viability.

In line with our data, Sun *et al*. [25] have found that verapamil concentrations of 6–10 µmol/L can completely reverse MDR in malignant tumour cells by inhibiting PGP and enhancing tumour cell sensitivity to chemotherapy; however, unfortunately, when serum verapamil concentration reaches 1–2 µmol/L, cardiac toxicity limits the drug’s clinical application. Nevertheless, as verapamil undergoes extensive hepatic first pass metabolism [26], it is theoretically feasible that the side effect of the drug can be avoided by using targeted intra-hepatic injections in combination with chemoembolisation. According to recent experimental reports, the efficacy and safety of such protocols are promising in patients with HCC [27]–[28].

In conclusion, our study shows that the resistance of HCC cells to multi-targeted tyrosine kinase inhibitors such as sorafenib and sunitinib is mediated by lysosomal sequestration (which is particularly evident in HCC cells with epithelial features), a mechanism that can be modulated by MDRP blockers such as verapamil. Our findings therefore pave the way for *in vivo* studies aimed at exploring the feasibility of the sequential administration of tyrosine kinase inhibitors and verapamil as a means of improving the chemosensitivity of liver cancer.

## Supporting Information

S1 Figure NH_4_Cl cell treatment. Representative pictures of cell lines cultured with NH_4_Cl 3 mM for 30 minutes (A) followed by sunitinib 12 µM+NH_4_Cl 3 mM for further 90 minutes (B) (Well 1) or with sunitinib12 µM for 60 minutes (C) followed by sunitinib 12 µM+NH_4_Cl 3 mM for further 90 minutes (D) (well 2). Original magnification 20x.
(TIF)

S2 Figure MTT assay with sunitinib and NH_4_Cl. Both HepG2 and SNU475 cell lines showed a decreased IC_50_ when they were pre-incubated with sunitinib or NH_4_Cl as compared to the treatment with sunitinib alone (P<0.05 and P<0.01, respectively). (SUN = sunitinib alone; PRE NH_4_Cl = pre-incubation with NH_4_Cl; PRE SUN = pre-incubation with sunitinib).
(TIF)

S1 Video Time-lapse experiments. Time-lapse video of HepG2 cells loaded with sunitinib 12 µM for two hours before being transferred to a Nikon Biostation IM. The pictures were taken every 10 minutes for seven hours. Original magnification 20x.
(ZIP)
