# Supplementary figures and images for: Giant Lysosomes as a Chemotherapy Resistance Mechanism in Hepatocellular Carcinoma Cells
Source: PLoS One. 2014 Dec 10;9(12):e114787. doi: 10.1371/journal.pone.0114787 (PMC4262459; doi:10.1371/journal.pone.0114787)

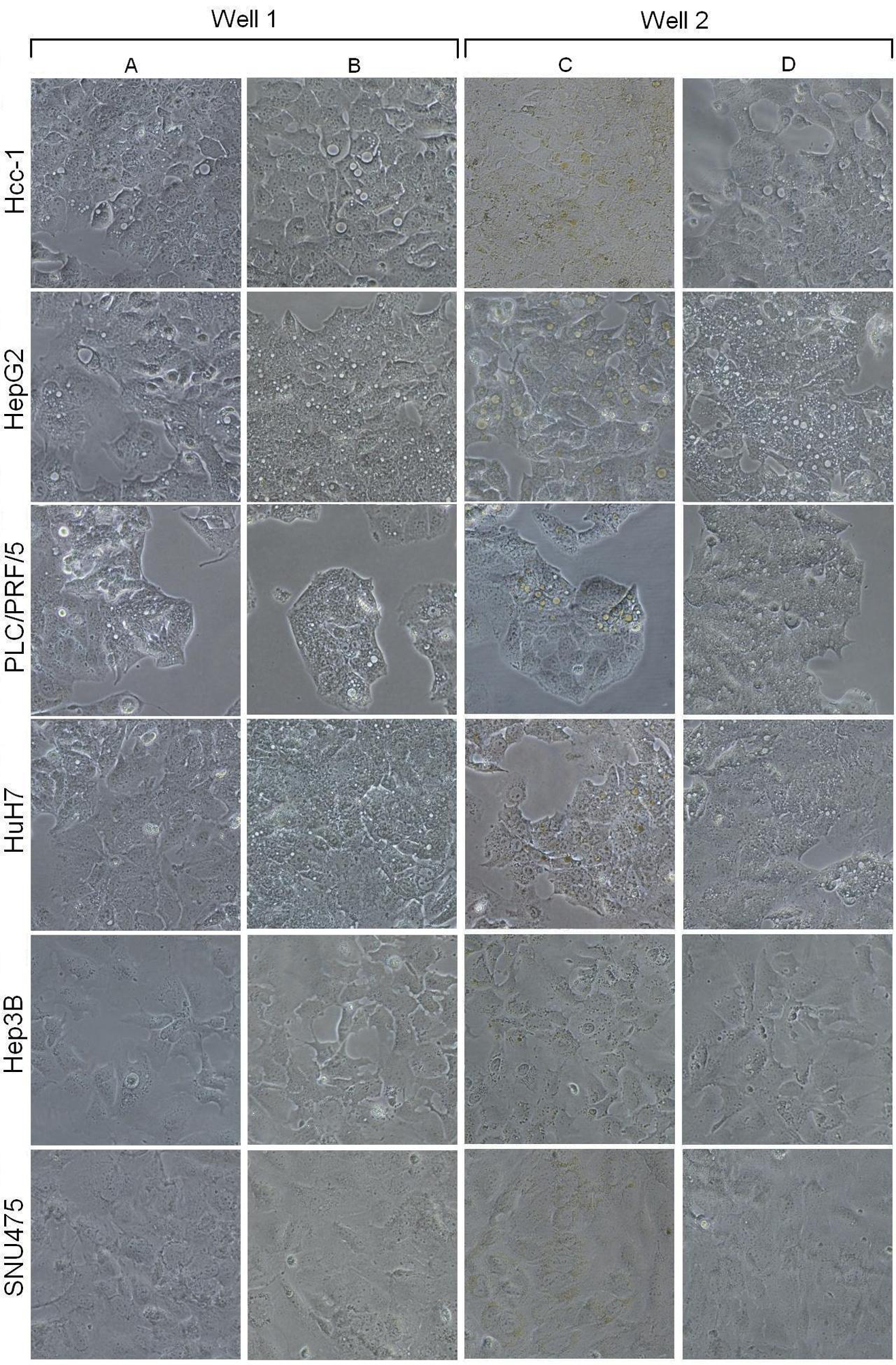

Supplement: S1 Figure — NH4Cl cell treatment. Representative pictures of cell lines cultured with NH4Cl 3 mM for 30 minutes (A) followed by sunitinib 12 µM+NH4Cl 3 mM for further 90 minutes (B) (Well 1) or with sunitinib12 µM for 60 minutes (C) followed by sunitinib 12 µM+NH4Cl 3 mM for further 90 minutes (D) (well 2). Original magnification 20x. (TIF) [file pone.0114787.s001.tif]

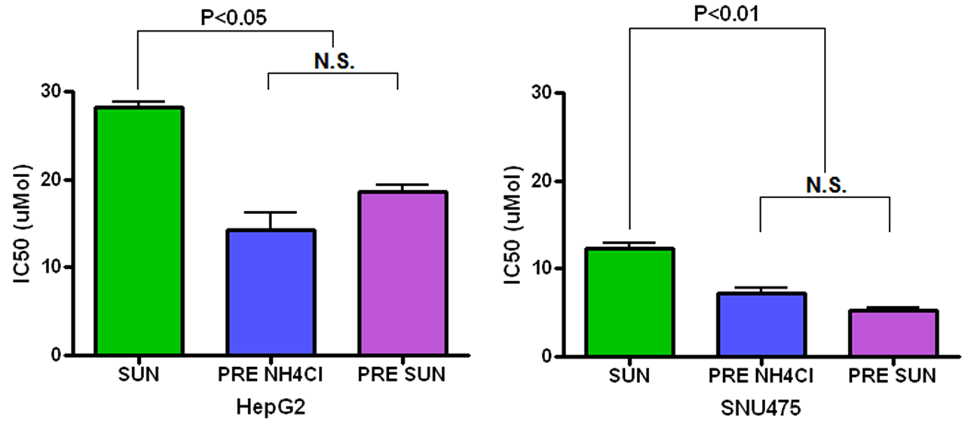

Supplement: S2 Figure — MTT assay with sunitinib and NH4Cl. Both HepG2 and SNU475 cell lines showed a decreased IC50 when they were pre-incubated with sunitinib or NH4Cl as compared to the treatment with sunitinib alone (P<0.05 and P<0.01, respectively). (SUN = sunitinib alone; PRE NH4Cl = pre-incubation with NH4Cl; PRE SUN = pre-incubation with sunitinib). (TIF) [file pone.0114787.s002.tif]
